# Association between dietary patterns and prediabetes, undetected diabetes or clinically diagnosed diabetes: results from the KORA FF4 study

**DOI:** 10.1007/s00394-020-02416-9

**Published:** 2020-10-30

**Authors:** Giulia Pestoni, Anna Riedl, Taylor A. Breuninger, Nina Wawro, Jean-Philippe Krieger, Christa Meisinger, Wolfgang Rathmann, Barbara Thorand, Carla Harris, Annette Peters, Sabine Rohrmann, Jakob Linseisen

**Affiliations:** 1 Division of Chronic Disease Epidemiology, Epidemiology, Biostatistics and Prevention Institute, University of Zurich, Zurich, Switzerland; 2 Independent Research Group Clinical Epidemiology, Helmholtz Zentrum München, German Research Center for Environmental Health, (GmbH), Ingolstädter Landstr. 1, 85764 Neuherberg, Germany; 3 Chair of Epidemiology, Ludwig-Maximilians-Universität München, UNIKA-T Augsburg, Augsburg, Germany; 4 Institute for Biometrics and Epidemiology, German Diabetes Center, Leibniz Center for Diabetes Research at Heinrich Heine University Düsseldorf, Düsseldorf, Germany; 5 German Center for Diabetes Research (DZD E.V.), Neuherberg, Germany; 6 Institute of Epidemiology, Helmholtz Zentrum München, German Research Center for Environmental Health (GmbH), Neuherberg, Germany; 7 Division of Metabolic and Nutritional Medicine, Dr. Von Hauner Children’s Hospital, University of Munich Medical Center, Munich, Germany

**Keywords:** Dietary patterns, Glucose tolerance status, Prediabetes, Type 2 diabetes, Undetected diabetes, Western pattern

## Abstract

**Purpose:**

Diet is one of the most important modifiable risk factors for the development of type 2 diabetes. Here, we aim to identify dietary patterns and to investigate their association with prediabetes, undetected diabetes and prevalent diabetes.

**Methods:**

The present study included 1305 participants of the cross-sectional population-based KORA FF4 study. Oral glucose tolerance test (OGTT) measurements together with a physician-confirmed diagnosis allowed for an accurate categorization of the participants according to their glucose tolerance status into normal glucose tolerance (*n* = 698), prediabetes (*n* = 459), undetected diabetes (*n* = 49), and prevalent diabetes (*n* = 99). Dietary patterns were identified through principal component analysis followed by hierarchical clustering. The association between dietary patterns and glucose tolerance status was investigated using multinomial logistic regression models.

**Results:**

A Prudent pattern, characterized by high consumption of vegetables, fruits, wholegrains and dairy products, and a Western pattern, characterized by high consumption of red and processed meat, alcoholic beverages, refined grains and sugar-sweetened beverages, were identified. Participants following the Western pattern had significantly higher chances of having prediabetes (odds ratio [OR] 1.92; 95% confidence interval [CI] 1.35, 2.73), undetected diabetes (OR 10.12; 95% CI 4.19, 24.43) or prevalent diabetes (OR 3.51; 95% CI 1.85, 6.67), compared to participants following the Prudent pattern.

**Conclusion:**

To our knowledge, the present study is one of the few investigating the association between dietary patterns and prediabetes or undetected diabetes. The use of a reference group exclusively including participants with normal glucose tolerance might explain the strong associations observed in our study. These results suggest a very important role of dietary habits in the prevention of prediabetes and type 2 diabetes.

**Electronic supplementary material:**

The online version of this article (10.1007/s00394-020-02416-9) contains supplementary material, which is available to authorized users.

## Introduction

Type 2 diabetes is one of the leading causes of morbidity and mortality in the developed world, representing a major public health issue and placing an heavy and increasing financial burden to the health-care system of different countries [1, 2]. The prevalence of diabetes increased rapidly over the past decades, and the global number of individuals living with diabetes is expected to increase from 451 million in 2017 to 693 million by 2045 [1]. Additionally, the disease is related to many complications, which potentially increase the risk of premature death [2]. In Europe, it is estimated that among all people with type 2 diabetes, 40% of the cases are undetected (i.e., blood glucose concentrations above the threshold for type 2 diabetes but never diagnosed by a physician) [1, 3]. Of high concern is also the increasing prevalence of prediabetes (i.e., blood glucose concentrations above normal but below the threshold for type 2 diabetes), as individuals with this condition are at increased risk of developing type 2 diabetes and other chronic diseases [1].

In addition to insufficient physical activity, unhealthy diet is considered as one of the most important modifiable risk factors for the development of type 2 diabetes [2]. These lifestyle behaviors are also related to increased overweight and obesity, which are well-known risk factors for the development of type 2 diabetes [1, 2]. Traditional approaches in nutritional epidemiology frequently investigate the association between diet and disease by focusing on single dietary components [4, 5]. However, dietary patterns might better capture the influence of diet on type 2 diabetes, as they can take the possible synergistic or antagonistic interactions of different nutrients present in foods into account [4, 5]. Dietary patterns represent the usual food consumption of an individual, which may help to better translate the findings into effective dietary recommendations [6, 7]. Moreover, the study of overall diets allow to consider dietary substitution or compensatory effects usually occurring with dietary changes [5].

The association between dietary patterns and the risk of type 2 diabetes has been previously investigated in several studies [6–13]. A Western dietary pattern, characterized by high consumption of red and processed meat, soft drinks, refined grains, fats and sweets, has frequently been associated with an increased disease risk [6–8, 11, 13]. On the other hand, a healthy or prudent dietary pattern, characterized by high consumption of fruits, vegetables, legumes, wholegrains and fish, has been associated with a modestly decreased risk of developing type 2 diabetes [7, 9, 12]. To our knowledge, however, the distinct association between dietary patterns and prediabetes or undetected diabetes has only been scarcely investigated in Western populations [14, 15]. Few other studies have been conducted in Asian populations [16–18], but the comparability of dietary patterns between culturally diverse populations is usually poor [4].

Using data from the Cooperative Health Research in the Region of Augsburg (KORA) FF4 study, our group previously identified significant associations between single food groups and prediabetes, undetected diabetes and prevalent diabetes [19]. To extend the understanding of these findings, the aims of the present study were to identify dietary patterns in the KORA FF4 study population and to investigate the association between dietary patterns and prediabetes, undetected diabetes and prevalent diabetes.

## Methods

The findings of the present study were reported according to standards of the “Strengthening the Reporting of Observational Studies in Epidemiology—Nutritional Epidemiology (STROBE-nut)” checklist [20].

### Study population

The analyses of the present study were performed using data from the cross-sectional population-based KORA FF4 study, which was conducted in the region of Augsburg in Southern Germany in 2013/2014. This is the second follow-up of the KORA S4 health survey, which was conducted between 1999 and 2001. Detailed information about the participation response has been previously published [21]. Briefly, of the 4261 individuals who participated in the KORA S4 survey, 2279 individuals also participated in the KORA FF4 study. During the visit at the study center, the participants answered self-administered questionnaires, participated in a computer-assisted face-to-face interview with trained study nurses and underwent a standardized physical examination, including anthropometric and blood pressure measurements, an oral glucose tolerance test (OGTT) and the collection of blood samples. Of the 2279 participants in the KORA FF4 study, individuals with type 1 diabetes (*n* = 6), unclear glucose tolerance status due to missing OGTT information (*n* = 93), missing dietary information (*n* = 638) and missing covariates (*n* = 2) were excluded from the present analyses. Additionally, participants with a diagnosis of cardiovascular disease (*n* = 82) or cancer (*n* = 153) were excluded, since having a severe disease may have led to changes in dietary behavior. A total of 1305 participants aged 38–87 years were eventually included in the present analyses. The characteristics of participants with and without dietary information are presented in Table S1.

The KORA FF4 study was approved by the Ethics Committee of the Bavarian Chamber of Physicians and all procedures followed the ethical standards of the Declaration of Helsinki. All participants provided written informed consent.

### Glucose tolerance status

Prevalent diabetes (i.e., clinically diagnosed diabetes) was defined by either self-reported diagnosis of type 2 diabetes or use of antidiabetic medication. The diagnosis was then further confirmed by the participant’s physician. All participants without a diagnosis of type 2 diabetes underwent a standard OGTT. The tests were conducted in the morning and participants were asked to fast for 10 h before the test, not to perform any heavy physical activity on the previous day and not to smoke before or during the test. Additionally, the OGTT were not performed in participants with medical contraindications. Fasting venous blood was collected for glucose measurement using serum tubes, before and 2 h after intake of 75 g of anhydrous glucose (Dextro OGT, Boehringer Mannheim, Germany). Serum glucose was analyzed using a hexokinase method (GLUFlex, Dade Behring, Deerfield, IL, USA) [22].

Participants were categorized by glucose tolerance status according to the diagnostic criteria of the American Diabetes Association (ADA) [23]. Normal glucose tolerance was defined as a fasting glucose concentration of < 5.6 mmol/l or a 2-h glucose OGTT concentration of < 7.8 mmol/l. Prediabetes was defined as impaired fasting glucose (fasting glucose concentration of 5.6–6.9 mmol/l), impaired glucose tolerance (2-h glucose OGTT concentration of 7.8–11.0 mmol/l), or a combination of both. Finally, a fasting glucose concentration of ≥ 7.0 mmol/l or a 2-h glucose OGTT concentration of ≥ 11.1 mmol/l was considered as undetected diabetes.

### Dietary assessment

The dietary assessment of the KORA FF4 study consisted of up to three repeated 24-h food lists (24HFL) and one food frequency questionnaire (FFQ). A total of 1602 individuals completed at least one 24HFL and one FFQ. Of these individuals, 652 (40.7%) completed two and 826 (51.6%) completed three 24HFL. As closed lists, the 24HFL included > 300 food items and were used to assess food consumption over the past 24 h. Additionally, the 24HFL also included information about intake of dietary supplements [24]. The FFQ, which was based on the German multilingual European Food Propensity Questionnaire (EFPQ), included 148 food items and was used to determine the frequency and amount of consumption over the past year [25].

Habitual food intake was computed using an advanced blended two-step approach as described in detail elsewhere [26], and further categorized into different food groups according to the classification system of the European Prospective Investigation into Cancer and Nutrition Software (EPIC-Soft) [27]. Additionally, the participants’ nutrient intake was estimated by linking the habitual food intake data to the National Nutrient Database (Bundeslebensmittelschlüssel BLS 3.02). To facilitate identification of dietary patterns, the food groups were rearranged into 23 food categories based on previous literature [19, 28]. A detailed description of the food categories is presented in Table S2.

### Assessment of dietary patterns

Dietary patterns were identified through principal component analysis and clustering in a two-step approach, analogously to Krieger and colleagues [29]. First, the 23 food categories were standardized by total energy intake and expressed in g/1000 kcal. Principal component analysis was then applied to the energy-standardized food consumption. Since extreme energy intake values led to extreme values in the energy-standardized food consumption, individuals below the 1st and above the 99th percentiles of energy intake (*n* = 34) were considered as supplementary individuals for the principal component analysis (i.e., they were not used to build principal components). In the present analysis, seven principal components were retained based on multiple criteria, including eigenvalues > 1, scree plot and total explained variance (Fig. S1). The retained principal components were subsequently used as input to hierarchical clustering using the Ward’s criterion [30], and the partitioning was further consolidated using a k-means clustering algorithm. The number of clusters to retain was determined using the decrease in within-inertia from *n* to *n* + 1 cluster (Fig. S2) as well as partition interpretability. Two clusters were eventually retained for interpretation. The stability of the dietary patterns was further tested by using five principal components as input for hierarchical clustering. Very similar clusters were obtained.

### Assessment of covariates

Potential covariates were selected according to the previous literature on the association between diet and type 2 diabetes [19, 28]. Age (years), sex (male/female), marital status (single/married/divorced/widowed), education (< 10 years/10–12 years/≥ 13 years, in accordance with the German education system), physical activity (active ≥ 1 h per week in at least one season (summer or winter)/inactive) [31, 32], smoking status (never/former/current) and hypertension (blood pressure ≥ 140/90 mmHg or use of hypertensive medication, yes/no) were assessed during computer-assisted face-to-face interviews by trained investigators or through self-administered questionnaires. Waist circumference (cm), body mass index (BMI, kg/m^2^), blood pressure (mmHg), total cholesterol (mmol/l) and triglycerides (mmol/l) were measured at the study center by trained personnel according to international standard protocols. Waist circumference was measured to the nearest 0.1 cm at the minimum abdominal girth. Body weight and body height, used to calculate the BMI, were measured in light clothing to the nearest 0.1 kg and 0.5 cm, respectively. Finally, measurements of blood pressure were performed three times with participants in a sitting position using an automatic and validated device [33].

### Statistical analysis

Descriptive statistics were used to characterize the KORA FF4 participants overall and by dietary patterns. Odds ratio (OR) and 95% confidence intervals (CI) were calculated by multinomial logistic regression models to investigate the association between dietary patterns and glucose tolerance status (i.e., normal glucose tolerance, prediabetes, undetected diabetes and prevalent diabetes). Three different regression models were fitted. Model 1 was adjusted for age and sex. Model 2 was further adjusted for marital status, education, physical activity and smoking. Marital status was added as adjusting factor in the regression models because of the significant associations with both diet and health outcomes observed in previous studies [34, 35]. Finally, because of their potential mediation role in the association between diet and type 2 diabetes, the variables waist circumference, hypertension, total cholesterol and triglycerides were added to a separate model (model 3). Moreover, to highlight the difference in results obtained when using OGTT measurements for glucose tolerance status and to enhance comparability with previous literature, a further analysis was conducted, in which multinomial logistic regression models were fitted considering participants with normal glucose tolerance, prediabetes and undetected diabetes altogether as reference group.

All statistical analyses were conducted with R software (version 3.5.3 for Windows). Principal component analysis and hierarchical clustering were performed using the *FactoMineR* package [36], whereas multinomial logistic regression models were fitted using the *nnet* package [37]. Statistical significance was set at 0.05 for all analyses.

## Results

**Fig. 1:**
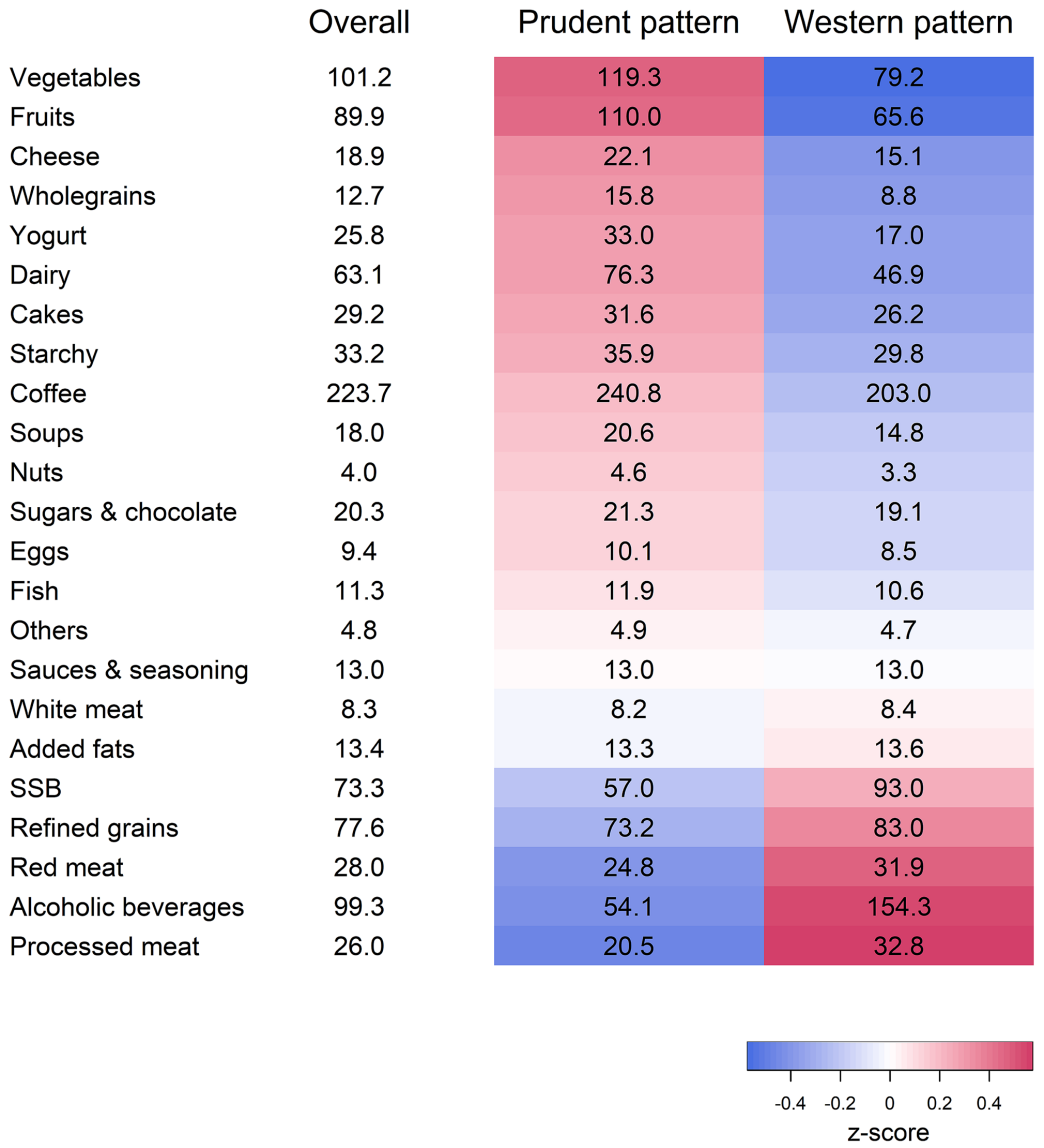
Energy-standardized food consumption (in g/1000 kcal) in the overall KORA FF4 population and in the identified dietary patterns. Colors indicate the mean of the z-standardized consumption of the food categories within one dietary pattern (i.e., the redder, the higher the consumption of the food category, the bluer, the lower the consumption of the food category compared to the mean consumption of the overall population); *SSB* sugar-sweetened beverages

Using principal component analysis and hierarchical clustering, we were able to identify two distinct dietary patterns among KORA FF4 participants, collectively explaining 52.7% of the total variation in diet (Fig. 1). The first pattern, labeled Prudent pattern, was characterized by high consumption of vegetables, fruits, wholegrains and dairy products (*n* = 707, 54.2%), while the second pattern, labeled Western pattern, was characterized by high consumption of processed meat, alcoholic beverages, red meat, refined grains and sugar-sweetened beverages (*n* = 598, 45.8%).

The characteristics of the KORA FF4 participants overall and by dietary patterns are presented in Table 1. Overall, 698 participants had a normal glucose tolerance, 459 had prediabetes, 49 had undetected diabetes and 99 had prevalent diabetes. Notably, important differences across dietary patterns were observed for the variables sex, BMI and waist circumference. In fact, 19.4% males, a BMI of 26.7 kg/m^2^ and a waist circumference of 99.5 cm in men and of 89.1 cm in women were observed among participants following the Prudent pattern, whereas 78.6% males, a BMI of 28.5 kg/m^2^ and a waist circumference of 103.0 cm in men and of 93.7 cm in women were observed among participants following the Western patterns. In addition, participants following the Prudent pattern were more likely to be older, physically active and never smokers.

**Table 1:** Characteristics of the KORA FF4 participants overall and by dietary patterns (*n* = 1305)

	Overall (*n* = 1305)	Prudent (*n* = 707)	Western (*n* = 598)
Sex, *n* (%)			
Males	607 (46.5)	137 (19.4)	470 (78.6)
Females	698 (53.5)	570 (80.6)	128 (21.4)
Age (years)	58.4 (11.6)	60.5 (11.8)	55.8 (10.9)
Marital status, *n* (%)			
Single	119 (9.1)	52 (7.4)	67 (11.2)
Married	956 (73.3)	504 (71.3)	452 (75.6)
Divorced	140 (10.7)	77 (10.9)	63 (10.5)
Widowed	90 (6.9)	74 (10.5)	16 (2.7)
Education, *n* (%) (years)			
< 10	69 (5.3)	50 (7.1)	19 (3.2)
10–12	743 (56.9)	383 (54.2)	360 (60.2)
≥ 13	493 (37.8)	274 (38.8)	219 (36.6)
BMI (kg/m^2^)	27.5 (4.9)	26.7 (4.7)	28.5 (5.1)
BMI categories, *n* (%)			
Underweight	6 (0.5)	4 (0.6)	2 (0.3)
Normal weight	427 (32.7)	286 (40.5)	141 (23.6)
Overweight	536 (41.1)	270 (38.2)	266 (44.5)
Obese	336 (25.7)	147 (20.8)	189 (31.6)
Waist circumference (cm)	95.7 (14.3)	91.2 (13.0)	101.0 (14.0)
Males	102.2 (12.3)	99.5 (10.0)	103.0 (12.8)
Females	90.0 (13.5)	89.1 (12.8)	93.7 (15.8)
Physical activity, *n* (%)			
Inactive	494 (37.9)	203 (28.7)	291 (48.7)
Active	811 (62.1)	504 (71.3)	307 (51.3)
Smoking status, *n* (%)			
Never	560 (42.9)	354 (50.1)	206 (34.4)
Former	558 (42.8)	284 (40.2)	274 (45.8)
Current	187 (14.3)	69 (9.8)	118 (19.7)
Hypertension, *n* (%)			
No	842 (64.5)	466 (65.9)	376 (62.9)
Yes	463 (35.5)	241 (34.1)	222 (37.1)
Glucose tolerance status, *n* (%)			
Normal glucose tolerance	698 (53.5)	433 (61.2)	265 (44.3)
Prediabetes	459 (35.2)	211 (29.8)	248 (41.5)
Undetected diabetes	49 (3.8)	17 (2.4)	32 (5.4)
Prevalent diabetes	99 (7.6)	46 (6.5)	53 (8.9)

Continuous variables are expressed as mean (SD); categorical variables are expressed as *n* (%)

BMI categories according to WHO standards (underweight: BMI < 18.5 kg/m^2^; normal weight: 18.5 ≤ BMI < 25.0 kg/m^2^; overweight: 25.0 ≤ BMI < 30.0 kg/m^2^; obese: BMI ≥ 30.0 kg/m^2^) [38]

*BMI* body mass index, *SD* standard deviation, *WHO* World Health Organization

Table 2 shows the results of multinomial logistic regression models investigating the association between dietary patterns and glucose tolerance status. In the multivariate adjusted model (model 2), participants following the Western pattern had significantly higher chances of having prediabetes (OR 1.92; 95% CI 1.35–2.73), undetected diabetes (OR 10.12; 95% CI 4.19–24.43) or prevalent diabetes (OR 3.51; 95% CI 1.85–6.67), compared to participants following the Prudent pattern. Further adjustment for waist circumference, hypertension, total cholesterol and triglycerides (model 3) attenuated the results to some extent (prediabetes: OR 1.50; 95% CI 1.03–2.18/undetected diabetes: OR 6.05; 95% CI 2.41–15.18/prevalent diabetes: OR 2.37; 95% CI 1.23–4.58).

**Table 2:** Association between dietary patterns and glucose tolerance status (*n* = 1305)

	Normal glucose tolerance		Prediabetes		Undetected diabetes		Prevalent diabetes	
	OR	95% CI	OR	95% CI	OR	95% CI	OR	95% CI
Western pattern	*n* = 265		*n* = 248		*n* = 32		*n* = 53	
Model 1	1	–	2.08	1.48–2.92	11.32	4.85–26.40	4.25	2.31–7.82
Model 2	1	–	1.92	1.35–2.73	10.12	4.19–24.43	3.51	1.85–6.67
Model 3	1	–	1.50	1.03–2.18	6.05	2.41–15.18	2.37	1.23–4.58

Reference: Prudent pattern (normal glucose tolerance: *n* = 433; prediabetes: *n* = 211; undetected diabetes: *n* = 17; prevalent diabetes: *n* = 46)

Model 1 adjusted for age, sex

Model 2 adjusted for age, sex, marital status, education, physical activity, smoking

Model 3 adjusted for age, sex, marital status, education, physical activity, smoking, waist circumference, hypertension, total cholesterol, triglycerides

*CI* confidence interval, *OR* odds ratio

To highlight the difference in results obtained when using OGTT measurements for glucose tolerance status and to enhance comparability with previous published studies, the association between dietary patterns and glucose tolerance status was also investigated considering participants with normal glucose tolerance, prediabetes and undetected diabetes altogether as reference group (Table 3). Overall, the analyses revealed a weaker association between dietary patterns and prevalent diabetes. In the multivariate adjusted model (model 2), participants following the Western pattern had a significantly higher chance of having prevalent diabetes (OR 2.06; 95% CI 1.14–3.75), compared to participants following the Prudent pattern. However, the association was no longer significant when the model was further adjusted for waist circumference, hypertension, total cholesterol and triglycerides (model 3, OR 1.55; 95% CI 0.85–2.84).

**Table 3:** Association between dietary patterns and glucose tolerance status considering participants with normal glucose tolerance, prediabetes and undetected diabetes altogether as reference group (*n* = 1305)

	Normal glucose tolerance/prediabetes/undetected diabetes		Prevalent diabetes	
	OR	95% CI	OR	95% CI
Western pattern	*n* = 545		*n* = 53	
Model 1	1	-	2.41	1.37–4.24
Model 2	1	-	2.06	1.14–3.75
Model 3	1	-	1.55	0.85–2.84

Reference: Prudent pattern (normal glucose tolerance/prediabetes/undetected diabetes: *n* = 661; prevalent diabetes: *n* = 46)

Model 1 adjusted for age, sex

Model 2 adjusted for age, sex, marital status, education, physical activity, smoking

Model 3 adjusted for age, sex, marital status, education, physical activity, smoking, waist circumference, hypertension, total cholesterol, triglycerides

*CI* confidence interval, *OR* odds ratio

## Discussion

Using data from the KORA FF4 study, we identified two distinct dietary patterns, a Prudent pattern and a Western pattern. Strong significant associations were observed between dietary patterns and glucose tolerance status. In fact, participants following the Western pattern, characterized by high consumption of red and processed meat, alcoholic beverages, refined grains and sugar-sweetened beverages, had significantly higher chances of having prediabetes, undetected diabetes and prevalent diabetes, compared to participants following the Prudent pattern.

To our knowledge, the present study is one of the few investigating the association between dietary patterns and prediabetes or undetected diabetes in a Western population [14, 15]. We relied on OGTT information to identify different groups of glucose tolerance status. The OGTT is an acute intervention to test the metabolic reaction in response to a defined glucose load. This test is considered the gold standard for the diagnosis of prediabetes or diabetes. HbA1c data provide less precise information as compared to OGTT data, especially for the definition of prediabetes [39, 40]. Day-to-day variation of the OGTT results has been described, and the clinical diagnosis of type 2 diabetes is ideally based on two OGTTs. However, in epidemiologic studies this additional effort is often not feasible. Mooy et al. have shown that random intra-individual variation in fasting and 2-h glucose concentrations had no distinct effect on the classification of new-onset diabetes. However, some variation was observed in the classification of individuals with prediabetes [41].

As expected, the association between dietary patterns and undetected diabetes observed in the present study was stronger compared to the association between dietary patterns and prevalent diabetes. This is likely due to reverse causation, since participants with prevalent diabetes were aware of the diagnosis and could have changed to a healthier dietary behavior before the beginning of the study. In contrast, the dietary habits of participants with undetected diabetes were more likely to remain unchanged, resulting in a potentially less biased estimate of the association between diet and type 2 diabetes.

Moreover, in all analyses, the association between dietary patterns and glucose tolerance status was attenuated after adjustment for waist circumference, hypertension, total cholesterol and triglycerides. However, since the association between diet and type 2 diabetes is likely to be mediated by these factors, adjustment for these variables may have led to an underestimation of the true effect. Unfortunately, due to the cross-sectional design of the study, the potential mediation effect of the above-mentioned variables could not be investigated in the present analyses.

The strong positive association between the Western pattern and glucose tolerance status observed in the present study may reflect a joint effect of single food groups. In fact, most of the food groups extensively consumed in the Western pattern were also associated with type 2 diabetes in our previous study [19], and in other studies [8, 11, 42–44]. A high consumption of red and processed meat has been frequently associated with increased chances of developing type 2 diabetes [8, 19, 42, 43]. Strong evidence also exists for the positive association between intake of sugar-sweetened beverages and type 2 diabetes [11, 19, 43]. Contrarily, results on the association between alcohol consumption and type 2 diabetes are rather mixed, with some studies observing a protective effect, especially with respect to moderate alcohol consumption [11, 43], and some observing a detrimental effect [19, 44]. Moreover, our Western pattern was also characterized by low consumption of several food groups that are frequently associated with a reduced type 2 diabetes risk, supporting the strong associations observed in the present analyses. In fact, high consumption of fruits and vegetables, wholegrains and dairy products has been consistently associated with a reduced risk of developing type 2 diabetes [43].

Despite the use of different methods, the dietary patterns identified in the present analysis share similar characteristics with dietary patterns identified in other Western populations, and our results are in line with previous studies. Using data of the large Nurses’ Health Study, Fung et al. observed a relative risk (RR) for extreme quintiles of 1.49 (95% CI 1.26–1.76) for a Western pattern generated by factor analysis [8], whereas Schulze et al. found an OR of 3.09 (95% CI 1.99–4.79) for a Western pattern generated by reduced rank regression, a method that allows to take into consideration different biomarkers potentially associated with the disease [11]. Similarly, an RR for extreme quintiles of 2.14 (95% CI 1.58–2.88) was observed by Malik et al. among participants of the Nurses’ Health Study II [6]. Moreover, also comparing extreme quintile of Western pattern adherence, van Dam et al. observed an RR of 1.59 (95% CI 1.32–1.93) using data of the Health Professionals Follow-up Study [7], and McNaughton et al. a hazard ratio (HR) of 2.27 (95% CI 1.67–3.11) in the Whitehall II Study [13].

The associations between dietary patterns and type 2 diabetes observed in the current study (undetected diabetes: OR 10.12; 95% CI 4.19–24.43/prevalent diabetes: OR 3.51; 95% CI 1.85–6.67) were stronger compared to most of the results in the previous literature. This might be due to the use of a reference group, which exclusively included participants with normal glucose tolerance. As mentioned before, the present study is one of the few investigating the association of dietary patterns with not only prevalent type 2 diabetes, but also prediabetes and undetected diabetes in a Western population [14, 15]. In fact, all KORA FF4 participants without a previous diagnosis of type 2 diabetes underwent an OGTT, which allowed for an accurate categorization of the participants according to their glucose tolerance status. However, these procedures are time consuming and expensive when conducted in large cohorts, and are therefore only rarely performed in such studies. Consequently, participants with prediabetes and undetected diabetes, who cannot be identified when relying on self-reports, are often included in the reference group [6–9, 11]. Despite the large burden, OGTT was conducted in some large cohort studies, and this allowed the categorization of participants with previously undetected diabetes as incident diabetes cases [10, 13, 45]. However, a distinction between individuals with normal glucose tolerance and prediabetes was usually not performed. Because individuals with prediabetes have an increased risk of developing type 2 diabetes and individuals with undetected diabetes already have type 2 diabetes [1], the true effect of diet on type 2 diabetes is likely to be stronger compared to what is estimated by the previous literature.

Few previous studies also investigated the association between dietary patterns and prediabetes [14, 16–18]. Generally, dietary patterns corresponding to a rather unhealthy diet were positively associated with prediabetes [14, 16, 18]. However, these studies derived different dietary patterns compared to the ones identified in the present study or were conducted in Asian populations, making a closer comparison with the results of our study difficult. The Western dietary pattern promotes derangement of glucose metabolism and different underlying mechanisms are being discussed. The most prominent seems to be that such a diet promotes systemic low-grade inflammation, similar to the effect of a high body fat mass [46]. The significant association between dietary patterns and prediabetes observed in our study (OR 1.92; 95% CI 1.35–2.73) suggests the need for dietary recommendations not only in individuals with prevalent type 2 diabetes, but also in individuals with prediabetes.

To enhance comparability between our study and the above-mentioned studies, we also conducted a further analysis, where individuals with normal glucose tolerance, prediabetes and undetected diabetes were considered altogether as the reference group. When adjusting for age, sex, marital status, education, physical activity and smoking, the analysis revealed an OR of 2.06 (95% CI 1.14–3.75) for prevalent diabetes among participants following the Western pattern compared to participants following the Prudent pattern. This result is very similar to the results of large cohort studies mentioned before, supporting the strong association observed in the main analysis of the present study. Further studies investigating the association between diet and type 2 diabetes should therefore consider a more accurate categorization of diabetes patients according to their glucose tolerance status whenever possible.

Major strengths of our study are the large sample size, which was originally randomly selected from the general population and had a high participation rate, the inclusion of a large variety of food items, the use of a sophisticated method to calculate dietary intake and the data collection by trained personnel. In addition, collection of OGTT measurements together with a physician-confirmed diagnosis allowed for an accurate categorization of the participants according to their glucose tolerance status and for the use of a reference group, which exclusively included participants with normal glucose tolerance. Another strength is the use of a two-step approach to derive dietary patterns. Like few others, we identified dietary patterns using principal component analysis followed by hierarchical clustering [29, 47, 48]. This approach enabled us to first remove the non-interpretable variation in diet and then to identify mutually exclusive dietary patterns based on the remaining interpretable variation [29, 47].

The present study has also some limitations. First, because of the cross-sectional design of the study, our results do not allow for drawing conclusions about the causal relationship between dietary patterns and glucose tolerance status. However, since participants with undetected diabetes were not aware of the diagnosis before the beginning of the study, it is unlikely that reverse causation influenced this association. Second, individuals who agreed to participate in the KORA FF4 study were probably more health-conscious compared to participants of the original KORA S4 health survey, potentially leading to participation bias. Also, under- or over-reporting and recall bias in dietary questionnaires cannot be excluded. Due to incomplete dietary assessment, a substantial part of the KORA FF4 study is lacking habitual dietary intake data. However, when comparing the characteristics of participants with and without dietary information, a high similarity between groups was found, also with respect to glucose tolerance status (Table S1). The methods used to identify dietary patterns have also some limitations. In fact, both principal component analysis and clustering methods partially involve subjective analytical decisions (e.g., number of dimensions or number of cluster to retain) and may therefore show limited stability or reproducibility [29]. However, very similar dietary patterns were identified when considering a different number of principal components as input for hierarchical clustering, suggesting stability of our dietary patterns. Moreover, the dietary patterns identified in the present study share several similarities with dietary patterns identified in other Western populations. Finally, given the important sex differences observed across dietary patterns, sex-stratified analyses could have been explored. However, the low number of participants in this study precluded the possibility to conduct analyses stratified by sex.

To conclude, a Prudent pattern and a Western pattern were identified among participants of the KORA FF4 study. The availability of OGTT measurements together with a physician-confirmed diagnosis allowed for an accurate categorization of the participants according to their glucose tolerance status. Strong, positive associations were observed between the Western pattern, characterized by high consumption of red and processed meat, alcoholic beverages, refined grains and sugar-sweetened beverages, and prediabetes, undetected diabetes or prevalent diabetes. The associations observed in the present study were stronger compared to the ones observed in our previous study, where diet was investigated using single food groups, suggesting that dietary patterns may be superior to individual food items for analyzing the association between diet and type 2 diabetes. These results suggest an important role of dietary habits in the prevention of prediabetes and type 2 diabetes, and may help to develop dietary recommendations that could serve as a basis for effective public health interventions, targeting not only individuals with type 2 diabetes, but also those at high risk of developing type 2 diabetes.

## Electronic supplementary material

Below is the link to the electronic supplementary material.

Supplementary file1 (PDF 253 kb)

## Data Availability

The datasets generated and/or analyzed during the current study are not publicly available due to national data protection laws and restrictions imposed by the ethics committee of the Bavarian Medical Association (“Bayerische Landesärztekammer”). The data that support the findings of this study are available from KORA (https://www.helmholtzmuenchen.de/en/kora-en/information-for-scientists/participating-in-kora/utilization-ofkora-data/index.html) but restrictions apply to the availability of these data, which were used under license for the current study. Data are however available upon reasonable request and with permission of KORA (https://www.helmholtzmuenchen.de/en/kora-en/information-for-scientists/participating-in-kora/utilization-ofkora-data/index.html).

## Code availability

Code is available upon reasonable request and approval.
