# Supplementary material for: Association between dietary patterns and prediabetes, undetected diabetes or clinically diagnosed diabetes: results from the KORA FF4 study
Source: Eur J Nutr. 2020 Oct 30;60(5):2331–41. doi: 10.1007/s00394-020-02416-9 (PMC8275503; doi:10.1007/s00394-020-02416-9)
Supplement: Supplementary file 1 — Supplementary file1 (PDF 253 kb) [file 394_2020_2416_MOESM1_ESM.pdf]

Electronic supplementary material

Association between dietary patterns and prediabetes, undetected diabetes or clinically diagnosed diabetes: results from the KORA FF4 study

Authors: Giulia Pestoni[1,2], Anna Riedl[2,3], Taylor A. Breuninger[2,3], Nina Wawro[2,3], Jean-Philippe Krieger[1], Christa Meisinger[2,3], Wolfgang Rathmann[4,5], Barbara Thorand[5,6], Carla Harris[6,7], Annette Peters[5,6], Sabine Rohrmann[1], Jakob Linseisen[2,3]

Affiliations:

[1]Division of Chronic Disease Epidemiology, Epidemiology, Biostatistics and Prevention Institute, University of Zurich, Zurich, Switzerland

[2]Independent Research Group Clinical Epidemiology, Helmholtz Zentrum München, German Research Center for Environmental Health, Neuherberg, Germany

[3]Ludwig-Maximilians-Universität München, Chair of Epidemiology, UNIKA-T Augsburg, Augsburg, Germany

[4]Institute for Biometrics and Epidemiology, German Diabetes Center, Leibniz Center for Diabetes Research at Heinrich Heine University Düsseldorf, Düsseldorf, Germany

[5]German Center for Diabetes Research (DZD e.V.), Neuherberg, Germany

[6]Institute of Epidemiology, Helmholtz Zentrum München, German Research Center for Environmental Health (GmbH), Neuherberg, Germany

[7]Division of Metabolic and Nutritional Medicine, Dr. von Hauner Children's Hospital, University of Munich Medical Center, Munich, Germany

Corresponding author:

Jakob Linseisen; Independent Research Group Clinical Epidemiology, Helmholtz Zentrum München, German Research Center for Environmental Health (GmbH), Ingolstädter Landstr. 1, 85764, Neuherberg, Germany; j.linseisen@helmholtz-muenchen.de; Tel. +49 89 3187-3202

Table S1: Characteristics of the participants with and without dietary information (after exclusions[#])

	Participants with dietary information (*n* = 1305)	Participants without dietary information (*n* = 524)
Glucose tolerance status, *n* (%)		
Normal glucose tolerance	53.5	50.4
Prediabetes	35.2	35.3
Undetected diabetes	3.8	4.4
Prevalent diabetes	7.6	9.9
Sex, *n* (%)		
Males	46.5	49.0
Females	53.5	51.0
Age (years)	58.4 (11.6)	59.6 (12.6)
Marital status, *n* (%)		
Single	9.1	12.4
Married	73.3	67.9
Divorced	10.7	9.9
Widowed	6.9	9.7
Education, *n* (%)		
< 10 years	5.3	9.8
10-12 years	56.9	59.2
≥ 13 years	37.8	31.0
BMI (kg/m^2)	27.5 (4.9)	28.3 (5.2)
Waist circumference (cm)	95.7 (14.3)	97.9 (13.8)
Physical activity level, *n* (%)		
Inactive	37.9	48.9
Active	62.1	51.1
Smoking status, *n* (%)		
Never	42.9	41.8
Former	42.8	38.2
Current	14.3	20.0
Hypertension, *n* (%)		
No	64.5	65.3
Yes	35.5	34.7

Continuous variables are expressed as mean (SD); categorical variables are expressed as *n* (%)

The number of participants with dietary information was obtained by applying all exclusion criteria used in the main analyses (i.e., number corresponds to participants eventually included in the study);

The number of participants with missing dietary information was obtained after applying all exclusion criteria used in the main analyses

Table S2: Description of food items included in the 23 food categories used to identify dietary patterns

Food categories	Included food items
Vegetables	Leafy, fruiting, root, cruciferous, cabbages, mushrooms, onions, garlic, stalk vegetables, sprouts, mixed salads and vegetables, legumes (peas, chickpeas, lentils, beans)<br>Except: vegetable juices, olives, soy products
Fruits	Fresh fruits, dried fruits, stewed fruits, mixed fruits, olives<br>Except: fruit juices
Nuts	Nuts (including nut spreads), seeds
Dairy products	Milk, milk beverages, cream desserts, puddings (milk-based), cream, milk for coffee and creamers
Yogurt	Nature yogurt, fruit yogurt, kefir
Cheese	Hard cheese, fresh cheese, soft cheese, cottage cheese, quark, mozzarella, feta cheese
Whole grains	Whole-grain bread, whole-grain toast, muesli
Refined grains	Flour, flakes, starches, semolina, pasta, rice, other grains, refined bread, crisp bread, rusks, breakfast cereals, salty biscuits, aperitif biscuits, crackers, dough and pastry (puff, shortcrust, pizza)
Starchy	Potatoes, potato products
Red meat	Fresh meat and offal (beef, veal, pork, lamb/mutton, horse, goat), game
Processed meat	Sausages, smoked and cured meat, meat spreads; from red or white meat
White meat	Poultry
Fish	Fish, fish products, crustaceans, molluscs
Eggs	Eggs, egg products
Added fats	Vegetable oils, butter, margarines, baking and deep frying fats
Sugars & chocolate	Sugar, jam, marmalade, honey, syrup, chocolate and chocolate products, candy bars, paste, confetti, flocks, non-chocolate confectionery, nougat, cereal bars, ice cream, sorbet
Cakes	Cakes, pies, pastries, waffles, dry cakes, biscuits
SSB	Carbonated/soft/isotonic drinks, diluted syrups, fruit and vegetable juices, nectars
Coffee	Coffee
Alcoholic beverages	Beer, cider, wine, spirits, brandy, liqueurs, cocktails, punches
Sauces & seasoning	Tomato sauces, dressing sauces, mayonnaises and similar, condiments
Soups	Soups, bouillon
Others	Other unclassified products, soy products

SSB: sugar-sweetened beverages

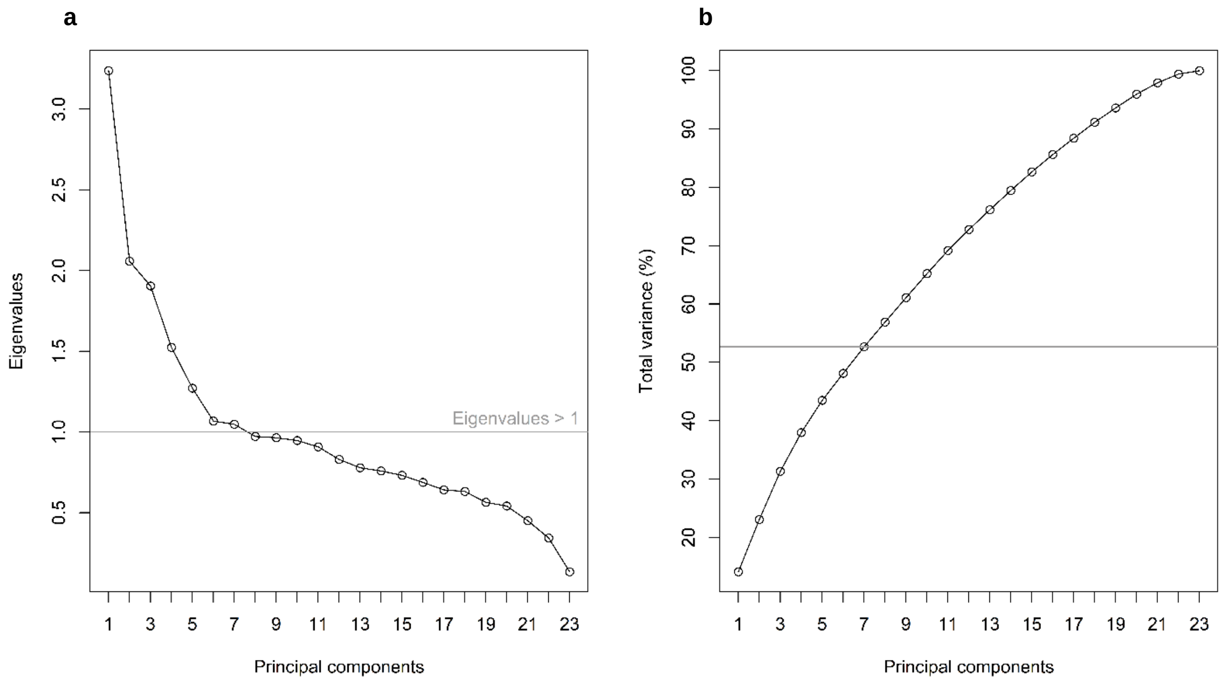

Fig. S1: Criteria used to determine the number of principal components to retain in principal component analysis. (a) Scree-plot, (b) Cumulative variance

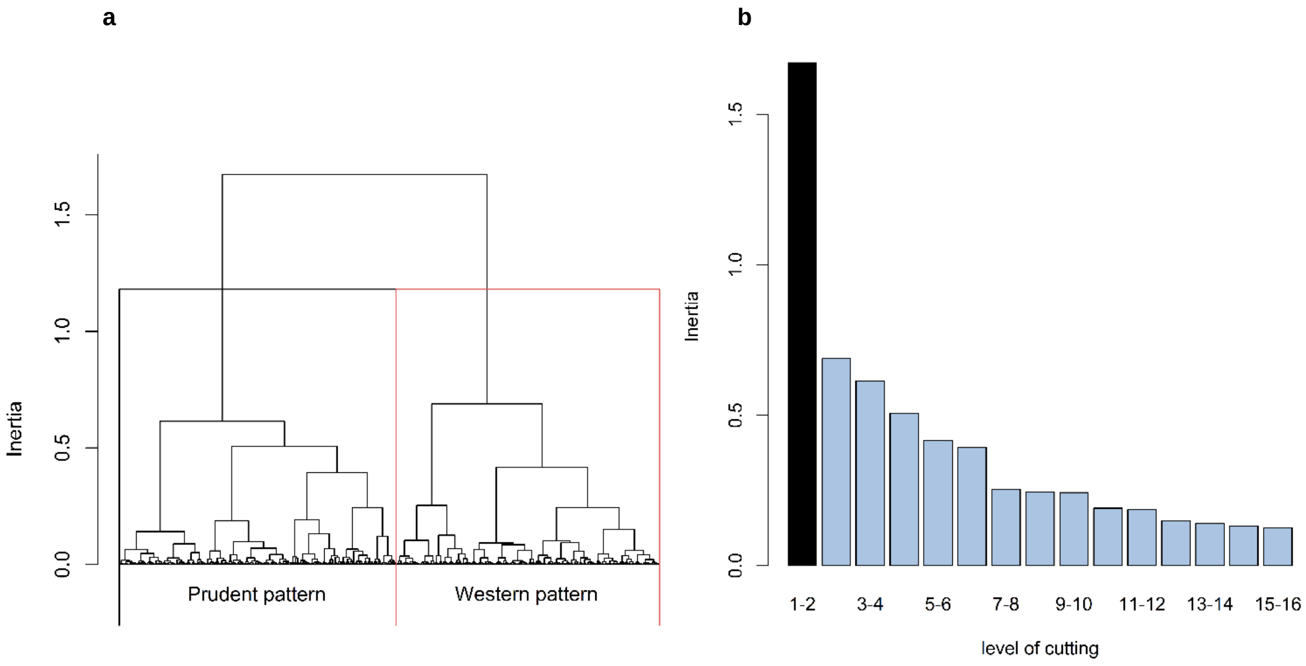

Fig. S2: Changes in inter-cluster inertia in hierarchical clustering. (a) Cluster dendogram by hierarchical clustering on the seven principal components obtained after principal component analysis, (b) Changes in inter-cluster inertia between partitions containing n or n+1 clusters
